# Characteristics of the clinical pharmacists' interventions at the main general tertiary care hospital in Qatar

**DOI:** 10.5339/qmj.2023.28

**Published:** 2023-10-28

**Authors:** Lina Naseralallah, Daoud Al-Badriyeh, Mounir Atchan, Palli Valappila Abdul Rouf, Moza Al Hail, Wessam El-Kassem, Dina Abushanab

**Affiliations:** Department of Pharmacy, Hamad Medical Corporation, Doha, Qatar. Email: dabushanab@hamad.qa ORCID iD: https://orcid.org/0000-0002-4162-8617; School of Pharmacy, College of Medical and Dental Sciences, University of Birmingham, Birmingham, UK; College of Pharmacy, QU Health, Qatar University, Doha, Qatar

**Keywords:** pharmacy, intervention, medication, medication-related problems, patient safety

## Abstract

Medication-related problems (MRPs) are prevalent throughout healthcare systems, whereby pharmacy-based interventions are pivotal to reducing occurrence. In the Middle East, including Qatar, the professional roles of pharmacists have been expanding to improve patient safety. This study aimed to characterize and analyze pharmacist-led interventions among hospitalized patients in the leading general hospital in Qatar. A retrospective analysis of pharmacist interventions in the internal medicine ward, critical care unit, and emergency department (ED) was conducted. Data were extracted from three periods of 1 month (March 1–31, 2018, July 15–August 15, 2018, and January 1–31, 2019). A descriptive type of analysis was undertaken. A total of 340 patients with 858 interventions were analyzed. The average age of the study participants was 51 years (SD ± 17.7). The study population was predominantly male (65%). The prevailing pharmacist intervention was adding drug therapy (27%), followed by medication discontinuation (18%) and dosage adjustments (16%). This pattern was maintained across all subpopulations, e.g., gender, age, and ward, except for the ED, where cessation of medication was the most frequent intervention (4%). The two pharmacological classes associated with most interventions were anti-infective and cardiovascular agents. Pharmacist interventions effectively identify, prevent, and resolve MRPs in general inpatient settings in Qatar.

## Introduction

Patient safety is a core objective of several national and international healthcare systems.^1,2^ Since the release of “To Err Is Human” by the Institute of Medicine (IOM), tremendous global efforts have been dedicated to ensuring the provision of optimum care to patients and minimizing the occurrence of medication-related problems (MRPs).^3^ The Pharmaceutical Care Network Europe (PCNE) has defined MRPs as “an event or circumstance involving drug therapy that actually or potentially interferes with desired health outcomes.”^4^ The PCNE classified the causes of MRPs into nine domains: drug selection, drug form, dose selection, treatment duration, dispensing, drug use process, patient-related, patient transfer-related, and others, e.g., no outcome monitoring.^4^ The most prevalent form of MRPs is medication errors, which could lead to patient harm, i.e., preventable adverse drug events (ADEs).^5^ Several studies reported that preventable ADEs are associated with economic burden, intensive care unit (ICU) admission, prolonged hospital stay, serious morbidity, and mortality.^5–8^ Various interventions have been described in the literature to reduce these incidents, including technology-driven, educational, and pharmacist-led interventions.^9–13^ For instance, clinical pharmacy-based interventions in the United States contributed to identifying and averting prescribing errors in 0.3%–1.9% of all medication orders.^13^

Most studies conducted in the Middle East have shown a positive influence of pharmacy interventions on health outcomes, including clinical, humanistic, and economic outcomes, by optimizing outpatient prescribing practices; however, only a few studies have focused on inpatient settings.^14–21^ In Qatar, there are two published attempts in relation to this aspect. The first focused on MRPs identified on discharge in four primary healthcare facilities, while the other focused on medication errors in neonatal intensive care units (NICUs).^22,23^ Aligning with international standards, the pharmacist role is substantially evolving in Qatar and is becoming more patient-centered. As the distribution of interventions varies between different settings, it is essential to investigate the clinical pharmacy interventions performed in the different settings and their characteristics.^14–21^ This is for a better understanding of the extent of the clinical pharmacist's role in preventing ADEs in the setting, helping in departmental planning and maximization strategies to harness the full potential of pharmacists. Therefore, in this study, we sought to describe and analyze the clinical pharmacist interventions to handle MRPs in patients hospitalized at the main general hospital in Qatar.

## Materials and methods

### Ethical Approval

The study was approved by the Medical Research Center (MRC), Hamad Medical Corporation (HMC) in 2019 (MRC-01-19-110). Given that the study is retrospective, the requirement for informed consent was waived. Anonymized data were collected to maintain confidentiality, and codes were used to cover identifiers.

### Study Design and Settings

This is a retrospective review study of pharmacist-documented interventions obtained from an Electronic Patient Record (EPR) system in Hamad General Hospital (HGH). HGH is the primary provider of secondary and tertiary healthcare in Qatar, with an approximate capacity of more than 600 beds.^22^ HGH is one of 14 hospitals under HMC, Qatar's public and leading healthcare provider. Pharmacy interventions are routine working-day tasks defined as “any action by a pharmacist that directly resulted in a change to patient management or therapy.”^23^ The patients' pharmacotherapeutic follow-up is performed through a daily review of medical records by the clinical pharmacists, including medications and laboratory tests, where the need for intervention can be identified. Intervention data are electronically embedded in an intervention sheet as part of the EPR system for clinical pharmacists to complete whenever an intervention occurs. No information is available about the onset of intervention after a medication order. This, however, can be between 5 and 120 minutes.

### Study Population

All adult patients admitted to the internal medicine general ward, critical care unit, and emergency department (ED) of HGH during the study follow-up duration were eligible for inclusion in the study. The study sample follow-up of three periods of 1 month, i.e., March 1–31, 2018, July 15–August 15, 2018, and January 1–31, 2019. We included all interventional recommendations by clinical pharmacists or clinical pharmacy specialists on hospitalized patients. The clinical interventions are, initially, only suggestions for consideration by physicians, requiring their approval to be implemented in patient cases. Thus, only interventions that physicians accepted were included in our study without conducting content or quality assessment, as long as the intervention was revised and approved by the respective prescriber. Interventions reported by non-clinical pharmacists (staff/operational pharmacists) were excluded from this study. Staff pharmacists work in outpatient or inpatient pharmacies to verify or dispense medications. In contrast, clinical pharmacists work in inpatient settings alongside other healthcare providers to develop healthcare plans for hospitalized patients. Missing or incomplete data from the intervention sheet were obtained from the EPR system of the patient.

### Outcomes

The primary outcome was to characterize clinical pharmacist interventions to prevent ADEs among patients admitted to the internal medicine, critical care, and emergency units of HGH. The interventions were categorized according to different characteristics (i.e., age, gender, medical disorder, pharmacological category, and hospital ward).

### Data Extraction and Synthesis

The intervention details and relevant sociodemographic data were abstracted from the EPR system into a spreadsheet. Patients were classified according to the hospital ward, gender, and age [adults (18–64 years old) and elderly (≥65 years old)].

To categorize the pharmacy clinical interventions, the authors developed a comprehensive data collection sheet that classifies interventions into 18 types to capture all the possible interventions that a clinical pharmacist could suggest.

### Sample Size

There are no standardized sample sizes for studies like the current one in the literature due to variations in the size of the setting and the prevalence of underlying conditions.^21,23–25^ Our sample size was based on duration, and we believe that over 25% of the year (hence, 3 out of 12 months) is a sufficient sample size. To enhance the representativeness of our sample size, we included interventions reported during three nonconsecutive months to cover the period immediately after the annual staff performance evaluation (first month of the year), before the annual evaluation (last month of the year), and the middle between these. This is because the evaluation may influence the documentation of interventions by clinical pharmacists, whereby they may become more vigilant.

### Statistical Analysis

Extracted data from patient records were populated into a data spreadsheet for descriptive analysis. The mean [± standard deviation (SD)] was calculated for continuous variables, while frequencies and percentages were calculated for categorical variables.

## Results

### Characteristics of the Study Patients

During the 3-month study period, 340 participants were admitted to HGH, with 858 clinical pharmacy interventions included in this study. The mean age of the population was 51 years, and 65.3% were male. The majority of patients were Arab (55.3%), followed by Asian (non-Arab) (34.4%), primarily admitted to the general internal medicine unit (53.8%), followed by the emergency unit (30.88%). Most interventions were retrieved around January 2019 (40.2%), 32.1% of interventions were between July and August 2018, and 27.7% were from March 2018. The demographic characteristics of the study population are presented in Table 1. Overall, the most commonly reported medical disorder was gastrointestinal (GI) disease (15.9%) and cardiovascular disease (CVD) (15%), followed by musculoskeletal disorders (MSD) at 12.8% and infectious diseases (ID) at 12.7%.

### Prevalence of the Different Types of Clinical Pharmacy Interventions

We reported clinical pharmacy interventions according to the classification sheet developed as part of this study and presented it in Table 2.

The most commonly performed intervention was the addition of medication (26.7%). This was followed by discontinuing medication (18.4%) and changing a medication dose (15.5%). The overall frequency of non-pharmacological interventions (i.e., addition adding laboratory tests, diagnostic tests, culture tests) was 4.6%, with the lowest being the addition of a culture test (0.2%). No interventions were observed in relation to a change in protocol. Among medication-related interventions, the rarest interventions were the decrease in medication duration (0.9%) and the addition of a vaccine (0.7%).

Consistent with the overall study population, the addition of another medication was the most frequent intervention in all subpopulations: adults (18.7%), elderly (8%), males (20.9%), and females (5.7%) (Table 2). The addition of a culture test was only identified in the elderly female group, while five out of six vaccine interventions were in adult male patients. Out of 50 formulation change interventions, 35 were from intraventricular (IV) to oral (PO) route, seven were from nasogastric tube (NGT) to PO, five were recommending an alternative therapy that is in the formulary (in cases where the physician prescribed a non-formulary medication), and one of each of PO to NGT, PO to different PO formulation, and PO to patch.

### Implicated Classes of Medications

**Figure 1.**
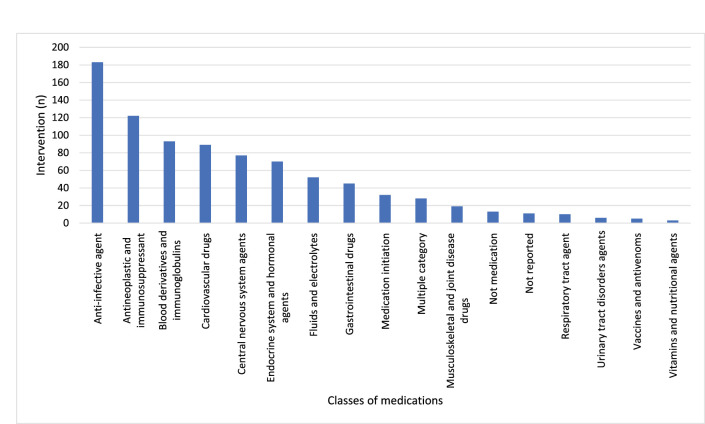
Classes of medications implicated in errors.

Of the 858 interventions, 183 (21.3%) errors were attributed to anti-infective agents before applying the intervention, including antibiotics, antivirals, antifungals, or antiparasitics (Figure 1).

Of these, the most common medications were piperacillin-tazobactam (*n* = 29), vancomycin (*n* = 22), ceftriaxone (*n* = 19), and meropenem (*n* = 16). Here, the most reported interventions were discontinuing medication (20.8%) and decreasing the dose (15.9%). Only two interventions recommended a decrease in the treatment duration. Appendix 1 presents the distribution of interventions according to the medication classes.

Cardiovascular drugs were the second most common class of medications associated with errors (*n* = 122, 14.2%), with enoxaparin being the top identified agent (*n* = 27), followed by 13 interventions for both dalteparin and unfractionated heparin (UFH). Discontinuation of medication was reported in 17.2% of interventions under this drug category, followed by the addition of a prophylactic agent (16.4%).

Around 6% of interventions were related to endocrine agents, where insulin was the highest single agent associated with interventions (*n* = 39). Correspondingly, only 0.7% and 1.1% of the overall interventions were associated with vaccine recommendations and blood derivatives.

After implementing the clinical pharmacy interventions, the same trend of pharmacological class involvement was maintained, except that the gap was smaller between anti-infective agents (*n* = 152) and cardiovascular drugs (*n* = 140).

### Implicated Hospital Wards

#### General Internal Medicine Inpatient Units

Of the 858 interventions, 459 (53.5%) were from clinical pharmacists allocated to general internal medicine units. Adding another medication (14.5%) and discontinuing medication (9.8%) were the two highest reported types of change in resource use. The decrease in medication dose (3.6%) was shown to be the third most common type of change in resource use, followed by 28 interventions for each increase in medication dose and the addition of a prophylactic agent. There was only one intervention for the addition of a vaccine and a culture test. Four out of seven documented additions of diagnostic test interventions occurred in this group of patients (Table 2).

Tables 3A shows the distribution of interventions according to hospital ward, based on age. Tables 3B shows the distribution according to hospital ward, based on gender. A similar trend was detected in the subgroups of adult, elderly, male, and female. However, adding a prophylactic agent during hospitalization in the elderly and females was more common than dose adjustments. The distribution of interventions according to the medication classes in patients admitted to internal medicine wards was similar to that observed in the overall study population and is presented in Appendix 2.

#### Critical Care Inpatient Units

Of the 858 interventions, 253 (29.5%) occurred in critical care units. The two most frequent types of change in resource use were maintained in this cohort of patients, as they were in the internal medicine population (Table 2). Unlike the interventions obtained from general internal medicine units, the increase in medication dose (4%) was more common than the decrease in the dose (2.5%). Only five interventions were about the addition of a prophylactic agent during hospitalization, and two were for each of the decrease in medication duration and the addition of a vaccine, while only one intervention reported an addition of a culture test.

Even though the same pattern of interventions in the general study population was maintained in the adult and female groups, the number of interventions recommending increasing medication dose exceeded the number of interventions recommending discontinuing medication in the elderly and male groups (Tables 3A and 3B). Anti-microbial agents were most commonly associated with interventions (*n* = 57) in critical care units; however, fluids and electrolytes (*n* = 32) surpassed the cardiovascular medications (*n* = 25) (Appendix 2).

### Emergency Department (ED)

Clinical pharmacists working in the ED recorded 146 (17.0%) interventions out of 858 total included interventions in this study. Distinct from the order of interventions prevalence observed in the overall study population and all previously discussed subcategories, the prevailing intervention in the ED was discontinued medications (4.3%) (Table 2). The following types of change in resource use were the addition of another medication (3.9%), change in medication route (1.4%), decrease in dose (1.28%), and increase in dose (0.93%). Half of the vaccine recommendations were performed in the ED ward, and there were only two documentation instances of adding a prophylactic agent during hospitalization. Notably, most patients under this category were adults (*n* = 117) and males (*n* = 136), Tables 3A and 3B. Anti-infective agents continued to be the most commonly associated class with interventions (*n* = 28), followed by fluids and electrolytes (*n* = 20), cardiovascular medicines (*n* = 18), and GI medications (*n* = 14) (Appendix 2).

## Discussion

To our knowledge, this is the first study to describe clinical pharmacist-delivered interventions in an inpatient setting at a general tertiary hospital in Qatar. Findings from this study indicated that adding another medication was the most frequent intervention. This differs from previously published studies, which reported dose adjustment as the predominant intervention.^10,21,25^ However, only some of these studies included the addition of drug therapy in their classifications. Consistent with our results, one study conducted in outpatient clinics showed that pharmacists were actively involved in adjusting patients' therapies, which included prescribing medications.^26^ This adds a new dimension to pharmacist duties, including identifying untreated conditions. In most published studies conducted in various settings and at different levels of care, including the present one, discontinuation of inappropriate prescription and dose alterations were featured high in the intervention categories. This is expected given that clinical pharmacists are medication experts, and it is their responsibility to promote the rational use of medication.^10,20,25–27^

Similar to previous studies, most interventions pertained to anti-infective agents and cardiovascular medications, possibly due to the frequency of prescribing these agents.^21,25,28^ Adjusting dose and cessation of medicines were the most prevalent interventions for errors with the anti-microbial agents. This is justifiable since three of the four most frequently identified agents require renal dosage adjustment, i.e., piperacillin-tazobactam, vancomycin, and meropenem.^6^ Of particular interest in our findings is that stopping an anti-infective agent was the prevailing intervention under this pharmacological class. This suggests that more efforts are required to plan and implement anti-microbial stewardship programs, especially since the widespread reliance on ceftriaxone, the third top agent in our study, has been reported in the literature to be the significant driver of cephalosporin resistance.^29^

Among cardiovascular medications, the addition of a prophylactic agent during hospitalization was frequently reported, primarily due to the need for low molecular weight heparin (LMWH) and UFH as venous thromboembolism (VTE) prophylaxis. Although one would expect prophylaxis against VTE to be most prevalent in patients admitted to the ICU as they are at higher risk for VTE, our study showed that most prophylactic agents during hospitalization were added to patients in non-ICU settings. This could be due to the presence of ICU protocol in HGH and other hospitals worldwide that incorporated VTE prophylaxis as part of the initial assessment upon admission and in the follow-up daily rounds.^30^

Our results reinforce the importance of pharmacist-led medication reconciliation, a common practice in all hospitals under HMC, as medication initiation and discontinuation were frequently reported. Medication reconciliation programs are recognized as an effective method to tackle the burden of medication discrepancies and the subsequent potential patient harm that could occur during the transition of care.^11^

Even though the addition of a vaccine is expected in older patients as more vaccines are recommended to this age group, e.g., flu vaccine and pneumococcal polysaccharide vaccine, most vaccination recommendations in our study were in younger adults.^30^ This could be attributed to the presence of national-level vaccination campaigns and the home health service in Qatar that follows up with elderly patients and ensures that they are up-to-date with their vaccines.

As fluctuations in glucose levels are anticipated in hospitalized patients owing to stress and changes in medications and diet, insulin was the single agent with the highest frequency of intervention-requiring errors.^31^ The most prevalent intervention for insulin was the change in the prescribed dose.

Medications associated with interventions in ICU patients were distinct from what has been identified in the overall population. Fluids and electrolytes were the second most cited class. Previous studies elucidated our results by demonstrating that fluid and electrolyte imbalances are among the most encountered medical disorders in critically ill patients.^32^ Another noteworthy finding in our study was obtained from the ED, which reported medication discontinuation as the dominant intervention. This differs from other studies investigating interventions in the ED, where drug information inquiry and dosage modifications were more common. This implies that overprescribing, recognized as an international issue, could be happening at HGH ED, requiring close vigilance from ED clinical pharmacists.^33–35^

It is also noteworthy that the male and female subcategories needed to be more balanced. This, however, could be considered representative of Qatar's population as the latest demographical statistics in the country illustrated that approximately 76% of the population consisted of males.^36^

This study has limitations that should be acknowledged. The study design is retrospective, which has the inherent limitation of possibly less-than-perfect documentation of interventions. In addition, all interventions that satisfied the eligibility criteria were included without running a quality appraisal for their content, where we assumed that the interventions were inherently validated as they were reviewed by the intervening pharmacist and the physician who approved them. Overall, however, this study still provides crucial information on the prevalence and nature of MRPs and the extent of the clinical pharmacist's role in preventing ADEs. Additionally, findings from this research should be shared with prescribers to highlight the issue of ADEs, which will help them reflect on their current practices and subsequently take measures to reduce the occurrence of ADEs.

Overall, for future directions, there is a need for frequent clinical auditing of pharmacist interventions to ensure their quality and validity. Furthermore, maintaining, if not expanding, the pharmacist role with better coordination of care among healthcare professionals is necessary to ensure high-quality care to patients.

## Conclusion

This retrospective analysis showed that clinical pharmacist interventions effectively identify, prevent, and resolve MRPs in hospitalized patients at the main general hospital in Qatar. The prevailing pharmacist intervention was the addition of a medication, followed by medication discontinuation and dose adjustments. This pattern was maintained across all subcategories except for the ED, where cessation of medication was the most frequently identified.

## Author contributions

DA-B and DA: conceptualization, validation, and supervision. DA and DA-B: methodology. LN: Software and writing – original draft preparation. DA-B, LN, and MA: investigation. DA: resources and funding acquisition. MA: data curation. LN, DA-B, PA, MA, MA, WE, and DA: project administration and writing – review and editing. LN, DA-B, PA, MA, WE, and DA: visualization. All authors have read and agreed to the published version of the manuscript.

## Declaration of conflicting interest

The authors declare that there is no conflict of interest.

## Funding

This work was supported by the Medical Research Center (MRC) at Hamad Medical Corporation (HMC), grant number (MRC-01-19-110).

## Figures and Tables

**Table 1.** Demographic characteristics of the study population.

		**Male, *n* = 222**	**Female, *n* = 118**	**Total, *n* = 340**
Age (years) Mean ± SD		49.94 ± 17.61	56.75 ± 16.96	51.04 ± 17.66
Weight (kg) Mean ± SD		78.30 ± 18.96.79	74.80 ± 19.93	77.49± 26.63
Ward, no. (%)	General internal medicine	145 (65.32)	38 (32.20)	183 (53.82)
	Critical care	38 (17.12)	14 (11.86)	52 (15.3)
	Emergency	97 (43.69)	8 (6.78)	105 (30.88)
Date of intervention, no. (%)	March 2018	193 (22.49)	45 (5.24)	238 (27.73)
	July to August 2018	205 (23.89)	70 (8.16)	275 (32.05)
	January 2019	291 (33.93)	54 (6.29)	345 (40.22)
Comorbidities distribution according to interventions, no. (%)	Gastrointestinal (GI) disease	97 (11.31)	39 (4.54)	136 (15.85)
	Cardiovascular diseases (CVD)	96 (11.19)	33 (3.85)	129 (15.04%)
	Musculoskeletal disorders (MSD)	102 (11.89)	8 (0.93)	110 (12.82%)
	Infectious diseases (ID)	93 (10.96)	15 (1.75)	108 (12.71%)
	Others	301 (35.08)	74 (8.62)	375 (43.7)

**Table 2.** The distribution of pharmacy interventions according to age, gender, and hospital ward.

**Type of intervention**	**Age groups**		**Gender**		**Hospital ward**			**Total, *n* = 858 (%)**
	**Adult, *n* = 597 (%)**	**Elderly, *n* = 261 (%)**	**Male, *n* = 689 (%)**	**Female, *n* = 169 (%)**	**Emergency, *n* = 146 (%)**	**Critical care, *n* = 2 53 (%)**	**General internal medicine, *n* = 459 (%)**	
Addition of another medication	160 (18.65)	69 (8.04)	180 (20.98)	49 (5.71)	33 (3.85)	72 (8.39)	124 (14.45)	229 (26.69)
Discontinuation of a medication	109 (12.70)	49 (5.71)	125 (14.56)	33 (3.85)	37 (4.31)	37 (4.31)	84 (9.79)	158 (18.41)
Increase in medication dose	45 (5.24)	25 (2.91)	57 (6.63)	13 (1.52)	8 (0.93)	34 (3.96)	28 (3.26)	70 (8.15)
Decrease in medication dose	37 (4.31)	26 (3.03)	53 (6.18)	10 (1.16)	11 (1.28)	21 (2.45)	31 (3.61)	63 (7.34)
Change in medication route	38 (4.43)	12 (1.40)	38 (4.43)	12 (1.40)	12 (1.40)	17 (1.98)	21 (2.45)	50 (5.83)
Change in medication strength	35 (4.08)	7 (0.82)	33 (3.85)	9 (1.05)	7 (0.82)	8 (0.93)	27 (3.15)	42 (4.9)
Switching to alternative medication	30 (3.50)	11 (1.28)	35 (4.08)	6 (0.70)	6 (0.70)	12 (1.40)	23 (2.68)	41 (4.78)
Addition of a prophylactic agent	18 (2.10)	17 (1.98)	29 (3.38)	6 (0.70)	2 (0.23)	5 (0.57)	28 (3.26)	35 (4.08)
Therapeutic drug monitoring	23 (2.68)	8 (0.93)	28 (3.26)	3 (0.35)	7 (0.82)	7 (0.82)	17 (1.97)	31 (3.61)
Increase in medication duration	27 (3.15)	4 (0.46)	23 (2.68)	8 (0.93)	5 (0.57)	6 (0.70)	20 (2.34)	31 (3.61)
Addition of a lab test	23 (2.68)	7 (0.82)	23 (2.68)	7 (0.82)	6 (0.69)	7 (0.82)	17 (1.97)	30 (3.50)
Increase in medication frequency	19 (2.21)	9 (1.05)	23 (2.68)	5 (0.58)	2 (0.23)	11 (1.28)	15 (1.75)	28 (3.26)
Decrease in medication frequency	15 (1.75)	12 (1.40)	24 (2.8)	3 (0.35)	3 (0.35)	8 (0.93)	16 (1.87)	27 (3.15)
Decrease in medication duration	7 (0.82)	1 (0.12)	6 (0.71)	2 (0.23)	4 (0.48)	2 (0.23)	2 (0.23)	8 (0.94)
Addition of a diagnostic test	6 (0.70)	1 (0.12)	7 (0.82)	0 (0)	0 (0)	3 (0.35)	4 (0.47)	7 (0.82)
Addition of a vaccine	5 (0.58)	1 (0.12)	5 (0.58)	1 (0.12)	3 (0.35)	2 (0.23)	1 (0.12)	6 (0.70)
Addition of a culture test	0 (0)	2 (0.23)	0 (0)	2 (0.23)	0 (0)	1 (0.117)	1 (0.117)	2 (0.23)

**Table 3.** The distribution of pharmacy interventions according to age and gender versus hospital ward.

3A. The distribution of pharmacy interventions according to age versus hospital ward				
Change in resource use	Emergency	Critical care	General internal medicine	Total
**Adult; *n* (%)**	**117 (13.64)**	**142 (16.55)**	**338 (39.39)**	**597 (69.58)**
Addition of a diagnostic test	0 (0)	2 (0.23)	4 (0.47)	6 (0.7)
Addition of a lab test	5 (0.58)	6 (0.7)	12 (1.4)	23 (2.68)
Addition of a prophylactic agent	2 (0.23)	0 (0)	16 (1.86)	18 (2.1)
Therapeutic drug monitoring	5 (0.58)	6 (0.7)	12 (1.4)	23 (2.68)
Addition of a vaccine	3 (0.35)	2 (0.23)	0 (0)	5 (0.58)
Addition of another medication	25 (2.91)	41 (4.78)	94 (10.96)	160 (18.65)
Change in a medication route	10 (11.76)	12 (1.4)	16 (1.86)	38 (4.43)
Change in a medication strength	7 (0.82)	5 (0.58)	23 (2.68)	35 (41.18)
Decrease in a medication dose	6 (0.7)	10 (1.17)	21 (2.45)	37 (4.31)
Decrease in a medication duration	4 (0.47)	1 (0.12)	2 (0.23)	7 (0.82)
Decrease in a medication frequency	2 (0.23)	2 (0.23)	11 (1.28)	15 (1.75)
Discontinuation of a medication	30 (3.5)	21 (2.45)	58 (6.76)	109 (12.70)
Increase in a medication dose	8 (0.93)	14 (1.63)	23 (2.68)	45 (5.24)
Increase in medication duration	5 (0.58)	6 (0.7)	16 (1.86)	27 (3.15)
Increase in medication frequency	2 (0.23)	6 (0.7)	11 (1.28)	19 (2.21)
Switching to alternative medication	3 (0.35)	8 (0.93)	19 (2.21)	30 (3.50)
**Elderly; *n* (%)**	**29 (3.38)**	**111 (12.94)**	**121 (14.10)**	**261 (30.42)**
Addition of a culture test	0 (0)	1 (0.12)	1 (0.12)	2 (0.23)
Addition of a diagnostic test	0 (0)	1 (0.12)	0 (0)	1 (0.12)
Addition of a lab test	1 (0.12)	1 (0.12)	5 (0.58)	7 (0.82)
Addition of a prophylactic agent	0 (0)	5 (0.58)	12 (1.4)	17 (1.98)
Therapeutic drug monitoring	2 (0.23)	1 (0.12)	5 (0.58)	8 (0.93)
Addition of a vaccine	0 (0)	0 (0)	1 (0.12)	1 (0.12)
Addition of another medication	8 (0.93)	31 (3.61)	30 (3.50)	69 (8.04)
Change in medication route	2 (0.23)	5 (0.58)	5 (0.58)	12 (1.40)
Change in medication strength	0 (0)	3 (0.35)	4 (0.47)	7 (0.82)
Decrease in medication dose	5 (0.58)	11 (1.28)	10 (1.17)	26 (3.03)
Decrease in medication duration	0 (0)	1 (0.12)	0 (0)	1 (0.12)
Decrease in medication frequency	1 (0.12)	6 (0.7)	5 (0.58)	12 (1.40)
Discontinuation of a medication	7 (0.82)	16 (3.2)	26 (3.03)	49 (5.71)
Increase in medication dose	0 (0)	20 (2.33)	5 (0.58)	25 (2.91)
Increase in medication duration	0 (0)	0 (0)	4 (0.47)	4 (0.47)
Increase in medication frequency	0 (0)	5 (0.58)	4 (0.47)	9 (1.05)
Switching to alternative medication	3 (0.35)	4 (0.47)	4 (0.47)	11 (12.94)
3B. The distribution of pharmacy interventions according to gender versus hospital ward.				
Count of type of change in resources	Emergency	Critical care	General internal medicine	Total
**Female; *n* (%)**	**10**	**78**	**81**	**169**
Addition of a culture test	0 (0)	1 (0.12)	1 (0.12)	2 (0.23)
Addition of a lab test	0 (0)	4 (0.47)	3 (0.35)	7 (0.82)
Addition of a prophylactic agent	0 (0)	1 (0.12)	5 (0.58)	6 (0.7)
Therapeutic drug monitoring	1 (0.12)	0 (0)	2 (0.23)	3 (0.35)
Addition of a vaccine	0 (0)	0 (0)	1 (0.12)	1 (0.12)
Addition of another medication	7 (0.82)	20 (2.33)	22 (2.56)	49 (5.71)
Change in medication route	0 (0)	7 (0.82)	5 (0.58)	12 (1.40)
Change in medication strength	0 (0)	3 (0.35)	6 (0.7)	9 (1.05)
Decrease in medication dose	0 (0)	6 (0.7)	4 (0.47)	10 (1.17)
Decrease in medication duration	0 (0)	2 (0.23)	0 (0)	2 (0.23)
Decrease in medication frequency	0 (0)	2 (0.23)	1 (0.12)	3 (0.35)
Discontinuation of a medication	1 (0.12)	15 (1.75)	17 (1.98)	33 (3.85)
Increase in medication dose	1 (0.12)	8 (0.93)	4 (0.47)	13 (1.52)
Increase in medication duration	0 (0)	4 (0.47)	4 (0.47)	8 (0.93)
Increase in medication frequency	0 (0)	1 (0.12)	4 (0.47)	5 (0.58)
Switching to alternative medication	0 (0)	4 (0.47)	2 (0.23)	6 (0.7)
**Male; *n* (%)**	**136 (15.85)**	**175 (20.40)**	**378 (44.06)**	**689 (80.30)**
Addition of a diagnostic test	0 (0)	3 (0.35)	4 (0.47)	7 (0.82)
Addition of a lab test	6 (0.7)	3 (0.35)	14 (1.63)	23 (2.68)
Addition of a prophylactic agent	2 (0.23)	4 (0.47)	23 (2.68)	29 (3.38)
Therapeutic drug monitoring	6 (0.7)	7 (0.82)	15 (17.65)	28 (3.26)
Addition of a vaccine	3 (0.35)	2 (0.23)	0 (0)	5 (0.58)
Addition of another medication	26 (3.03)	52 (6.06)	102 (11.89)	180 (20.98)
Change in medication route	12 (1.40)	10 (1.17)	16 (1.86)	38 (4.43)
Change in medication strength	7 (0.82)	5 (0.58)	21 (2.45)	33 (3.85)
Decrease in medication dose	11 (1.28)	15 (1.75)	27 (3.15)	53 (6.18)
Decrease in medication duration	4 (0.47)	0 (0)	2 (0.23)	6 (0.7)
Decrease in medication frequency	3 (0.35)	6 (0.7)	15 (1.75)	24 (2.80)
Discontinuation of a medication	36 (42.35)	22 (2.56)	67 (7.81)	125 (14.57)
Increase in medication dose	7 (0.82)	26 (3.03)	24 (2.80)	57 (0.58)
Increase in medication duration	5 (0.58)	2 (0.23)	16 (1.86)	23 (2.78)
Increase in medication frequency	2 (0.23)	10 (1.17)	11 (1.28)	23 (2.68)
Switching to alternative medication	6 (0.7)	8 (0.93)	21 (2.45)	35 (4.08)

## APPENDICES

**Appendix 1.** The distribution of pharmacy interventions according to the medication class.

**Count of type of change in resources**	**ID**	**Anti-neoplastic and immunosuppressants**	**Blood derivatives and immunoglobulins**	**CVD**	**CNS**	**Endocrine system**	**Fluids and electrolytes**	**GI**	**Medication initiation**	**Multiple categories**	**MSD**	**Not medication**	**Not reported**	**Respiratory tract agent**	**Urinary tract disorder agents**	**Vaccines**	**Vitamins and nutritional agents**	**Total**
Addition of a culture test												2						2
Addition of a diagnostic test												7						7
Addition of a lab test												30						30
Addition of a prophylactic agent	4			20				1	9			1						35
Therapeutic drug monitoring (TDM)												30	1					31
Addition of a vaccine																6		6
Addition of another medication	20	1	2	16	2	14	40	26	79	2	5		1	6			15	229
Change in medication route	21	1		5	8	1	3	7		1	2						1	50
Change in medication strength	13	1	2	8	4	2	4			2				1			5	42
Decrease in medication dose	29	5		11	6	5	1	1		1	3						1	63
Decrease in medication duration	2	1	2	1			2											8
Decrease in medication frequency	4			2	6	1	1	4		1	6		1	1				27
Discontinuation of a medication	38	9	3	21	8	7	24	22	1	4	7		2	4	3		5	158
Increase in medication dose	16			16	5	18	11	1			1						2	70
Increase in medication duration	9		1	11		1	1	1	4		2						1	31
Increase in medication frequency	7	1		6	2	1	1	5			2			1			2	28
Switching to alternative medication	20			5	4	2	1	9										41
**Total**	**183**	**19**	**10**	**122**	**45**	**52**	**89**	**77**	**93**	**11**	**28**	**70**	**5**	**13**	**3**	**6**	**32**	**858**

*Abbreviations:* GI, Gastrointestinal disease; CVD, Cardiovascular diseases; CNS, Central nervous system; MSD, Musculoskeletal disorders; ID, Infectious diseases.

**Appendix 2.** Implicated medication classes in interventions, according to hospital ward.

**Class of medication**	**Emergency, *n* = 146**	**Critical, *n* = 253**	**General internal medicine, *n* = 459**	**Total, *n* = 858 (%)**
Anti-infective agent	28	57	98	183 (21.34)
Anti-neoplastic and immunosuppressants	5	8	6	19 (2.21)
Blood derivatives and immunoglobulins	3	2	5	10 (1.17)
Cardiovascular drugs	18	25	79	122 (14.22)
Central nervous system agents	11	10	24	45 (5.24)
Endocrine system and hormonal agents	10	21	21	52 (6.06)
Fluids and electrolytes	20	32	37	89 (10.37)
Gastrointestinal drugs	14	23	40	77 (8.97)
Medication initiation	10	28	55	93 (10.84)
Multiple categories	3	5	3	11 (1.28)
Musculoskeletal and joint disease drugs	5	8	15	28 (3.26)
Not medication	13	18	39	70 (8.16)
Not reported		1	4	5 (0.58)
Respiratory tract agents	1	7	5	13 (1.52)
Urinary tract disorder agents		1	2	3 (0.35)
Vaccines and antivenoms	3	2	1	6 (0.70)
Vitamins and nutritional agents	2	5	25	32 (3.73)
